# Potential role of IFN-γ and IL-5 in sepsis prediction of preterm neonates

**DOI:** 10.1515/med-2021-0206

**Published:** 2021-01-11

**Authors:** Jelena Vucic, Miodrag Vucic, Tatjana Stankovic, Hristina Stamenkovic, Sandra Stankovic, Dragan Zlatanovic

**Affiliations:** Department of Neonatology, Children’s Hospital, Clinical Center Nis, 18000 Nis, Serbia; Department of Malignant Hematological Disorders, Clinic of Hematology and Clinical Immunology, Medical Faculty, University of Nis, 18000 Nis, Serbia; Department of Immunology, Children’s Hospital, Clinical Center Nis, 18000 Nis, Serbia; Department of Endocrinology, Children’s Hospital, Clinical Center Nis, 18000 Nis, Serbia; Clinic for Physical Medicine and Rehabilitation, Medical Faculty, University of Nis, 18000 Nis, Serbia

**Keywords:** preterm neonates, sepsis, cytokines, prediction

## Abstract

Not fully maturated immune system in preterm neonates may contribute to the increased susceptibility to infection. The levels of some cytokines can be useful in the prediction and diagnosis of sepsis in premature neonates. In the present study, we evaluated the potential predictive role of IFN-γ and IL-5 in cord and venous blood, together with the determination of C-reactive protein and procalcitonin (PCT) for sepsis development in premature neonates. A total of 80 participants were included. The laboratory results and clinical histories showed that 21 participants had sepsis. Early onset sepsis was detected in 3 patients while late onset sepsis was observed in 18 participants. The venous plasma levels of IFN-γ and PCT was markedly increased in sepsis groups when compared to the participants without sepsis. On the other hand, levels of IL-5 did not significantly change in the evaluated groups of sepsis and in the control group of participants. Simultaneously, plasma venous levels were not altered in any of the evaluated groups. Obtained findings suggest that venous plasma levels of IFN-γ, rather than levels of IFN-γ in cord blood plasma, and PCT may have predictive potential for sepsis development in preterm neonates. Further studies are necessary to get more comprehension of the complex function of cytokines for sepsis development in preterm neonates.

## 1 Introduction

Globally, neonatal sepsis represents public health problem with high rate of morbidity and mortality. The most harmful effect is especially addressed to preterm neonates [1]. The health of preterm neonates deteriorates rapidly, causing septic shock, and neonates may die even before antimicrobial tests are ready [2]. Early onset sepsis (EOS) occurs in the first 72 h of life and it is usually caused by organisms transmitted vertically, from the mother to the infant before or at the time of birth. Late onset sepsis (LOS) develops after 72 h of life and may be induced by pathogens acquired by delivery or during the course of hospital care [3].

The diagnosis of neonatal sepsis is especially difficult. Primarily, unspecific signs and symptoms (tachypnea, thermal dysregulation, lethargy, abdominal distension and others) are often seen in clinical condition of neonatal sepsis [1]. The mechanisms of distinct neonatal response to infection is not clearly understood. However, pro- and anti-inflammatory cytokines have key roles and regulate the inflammation process and host response to infection. Although the immune system develops throughout the fetal period, it has been proved that different microbial antigens evoke innate and adaptive mechanisms of the host and release cytokines, resulting in clinical signs and outcomes [4].

Because inflammation has an important role in sepsis development, various studies have been conducted to evaluate inflammatory mediators which may permit an early diagnosis of sepsis. These reports observed potential role of IL-1β, IL-6, IL-8, TNFα and IL-10 in early sepsis diagnosis, by evaluating these cytokines in cord blood or in venous blood [5,6]. However, systemic review revealed that the role of inflammatory mediators varied, and additional studies are needed to clarify the role of mediators [7]. Therefore, in the current study, we tested whether the levels (in cord and venous blood) of some of the key cytokines of Th1 (IFN-γ) and Th2 (IL-5) phenotypes, may have predictive role in early diagnosis of sepsis in preterm neonates, along with some standard laboratory analysis.

## 2 Materials and methods

### 2.1 Study population

This prospective study was carried out from December 2017 to December 2018 in Pediatric Internal Diseases Clinic, Clinical Center of Nis, Serbia. The study population included 101 neonates born at 36 weeks of gestational age (GA). However, among premature neonates, 21 of them were excluded from the study due to maternal pregnancy-induced hypertension and premature rupture of membranes (PROMs). Also, we excluded from the study premature neonates with congenital malformations and laboratory-confirmed TORCH (Toxoplasma, rubella virus, cytomegalovirus, herpes simplex virus-II) as well as premature neonates born to mother with signs of infection (cervicovaginitis, chorioamnionitis, urinary tract infection and fever during delivery) or presence of any systemic disease. This study was approved by the Ethics Committee, Medical Faculty in Nis (35144/2017). Written informed consent was obtained from the parents for all recruited premature neonates, prior to study participation.

### 2.2 Definition of sepsis and study group allocation

Sepsis is defined as evidence of a systemic inflammatory response in the presence of suspected or proven infection. In our study, definition of neonatal sepsis was performed according to the Criteria published by International Pediatric Sepsis Consensus Conference [8].

All recruited participants were classified according to the infant’s age at birth into two groups (group 1: <32 weeks and group 2: 32–36 weeks of GA) [2]. Simultaneously, according to the time when the first sepsis episode occurred, both groups were divided into EOS when the first sepsis episode occurs <72 h of life and LOS when the first sepsis episode occurs >72 h of life [3]. Among the recruited participants, premature neonates without any sepsis episodes served as a control group [1].

### 2.3 Neonatal and maternal data

Neonatal and maternal data were retrospectively obtained from maternal and infant’s medical records. Weight, length of neonates, GA, sex, delivery mode, Apgar score at 1 min after birth, low growth for GA, patent ductus arteriosus (PDA), respiratory distress syndrome (RDS), periventricular, intraventricular hemorrhage, pregnancy-induced hypertension and PROM were obtained from hospital records. Low growth for GA was determined as weight and length less then 10th percentile for GA.

### 2.4 Blood samples and laboratory data

Immediately after delivery, we collected cord blood and venous blood samples by using tubes coated with K2-ethylenediaminetetraacetic acid (Sarstaedt, Numbrecht, Germany). Collected samples were centrifuged at 300 rpm for 10 min. After samples from all participants had been collected, the removed plasma was stored in sterile tubes at −80°C for later measurements of biomarker levels. Additionally, from collected venous blood, levels of C-reactive protein (CRP) was evaluated by using Horiba-Microsemi (Roma, Italy) system, and procalcitonin (PCT) was determined by using Cobas e411 system (Roche/Hitachi, Bellport, USA).

### 2.5 Measurement of cytokines

The concentrations of IFN-γ and IL-5 in venous and cord blood samples were detected simultaneously by using the ELISA technique, according to the manufacturer’s instructions. For this analysis, we used 96-well flat bottom plate kit Express Assay Format Human Cytokine Group (Bio-Rad, Hercules, USA). Further microplate reading was assessed by using BioPlex 2200 system (Bio-Rad, Hercules, USA), following the analytical methodology given by manufacturer’s instructions.

### 2.6 Statistical analysis

We evaluated the normality of the data by using Shapiro–Wilk and Kolmogorov–Smirnov tests. Nonnormal distributed data are expressed as minimum–maximum (median value) and comparisons between these groups were assessed using Kruskal–Wallis and/or Mann–Whitney test. Categorical variables are expressed as frequencies and compared by using the chi-square test. Normally distributed data are expressed as mean ± SD and compared by using ANOVA. All statistical tests were two sided and performed at a significance level of *p* = 0.05. Statistical analysis was performed by using SPSS Statistics in Windows environment.

## 3 Results

By fulfilling the inclusion criteria mentioned above, 80 preterm infants were included in the study of the 101 total infants collected during the examination period. Table 1 shows some clinical and demographic features of the mothers and preterm infants included in our study. As shown in Table 1, seven infants (8.75%) were born before 32 GA, while 73 infants (91.25%) were born between 32 and 36 GA. Further characteristics of study enrolled infants are shown in Table 1.

**Table 1:** Risk factors for neonatal sepsis

Risk factors	*n* (%), *N* = 101	*n* (%), *N* = 80
Prematurity – before 32 week of gestational age	8 (7.9%)	7 (8.75%)
Prematurity – 32–36 weeks of gestational age	93 (92.1%)	73 (91.25%)
Birth by cesarean section	40 (39.6%)	28 (35%)
Low weight for gestational age	12 (11.9%)	7 (8.75%)
Low growth for gestational age	10 (9.9%)	5 (6.3%)
Pregnancy-induced hypertension	14 (13.9%)	—
PROMs	9 (8.9%)	—
PDA	23 (22.8%)	22 (27.5%)
RDS	32 (31.7%)	31 (37.75%)
Periventricular and intraventricular hemorrhage	8 (7.9%)	7 (8.75%)

During follow-up, 21 of the 80 infants developed sepsis. Taking into account the GA of the infants, in group 1 (infants <32 weeks of GA), 2 infants (28.6%) developed EOS, 1 developed LOS (14.3%) and 4 infants (57.2%) did not develop sepsis. However, in group 2 (infants 32–36 weeks of GA), 8 infants (11%) demonstrated EOS, 10 infants (13.7%) with LOS, while 55 infants (75.3%) showed no septic episodes (Table 2). Comparing two groups (groups 1 and 2) with control group resulted in no significantly different incidence of sepsis development. Nevertheless, since in group 1 only three preterm infants developed sepsis, the results from this group should be considered, due to a small number of participants recruited in this group. Additionally, when two sepsis groups (EOS and LOS) were compared to the control group, the clinical variables birth weight, length, sex and Apgar score showed no statistical significance (*p* > 0.05). Also, the CRP concentration in venous blood plasma resulted in no statistical significance (*p* > 0.05) when compared to two sepsis (EOS and LOS) and control groups of premature infants (Table 2). On the other hand, PCT concentration in venous blood plasma showed statistical significance (*p* < 0.05) when EOS and LOS are compared as well as when LOS and control (with no sepsis) group (*P* < 0.001) are compared. Significantly higher (*p* < 0.001) PCT concentrations were observed when EOS and control group are compared (Table 2).

**Table 2:** Comparison of some clinical variables and levels of CRP and PCT among subjects by sepsis subgroups

	Early onset sepsis	Late sepsis	No sepsis	*P*
<32 GW	2 (28.6%)	1 (14.3%)	4 (57.2%)	0.256
32–36 GW	8 (11%)	10 (13.7%)	55 (75.3%)	
Normal growth for GA	11 (14.7%)	10 (13.3%)	54 (72%)	1
Low growth for GA	0 (0%)	0 (0%)	5 (100%)	
Female	5 (13.9%)	5 (13.9%)	26 (72.2%)	1
Male	5 (11.4%)	6 (13.6%)	33 (75%)	
Apgar score	4–9 (8)	8–9 (8)	4–9 (8)	0.227
CRP (mg/L)	0–15.4 (6.42)	0.5–5.3 (2.2)	0–55.8 (1.3)	0.082
PCT (ng/mL)	4.85–26.59 (10.43)	3.13–16.9 (6.66)	0–4.79 (1.18)	**Early vs late < 0.05**
				**Late vs no < 0.001**
				**Early vs late < 0.001**

**Figure 1:**
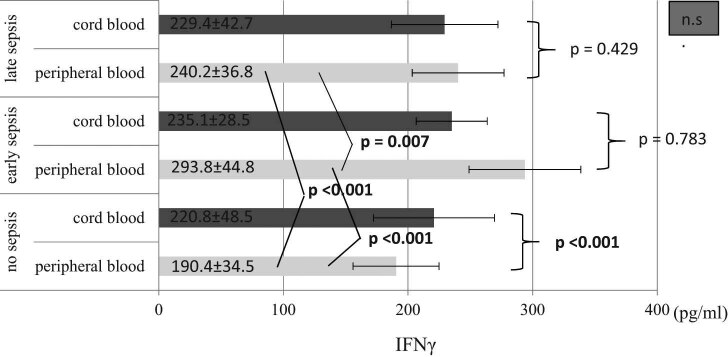
IFN-γ (pg/mL) levels in the cord blood and peripheral blood among sepsis subgroups and control group. The plasma levels of IFN-γ was evaluated as described in Material and methods section. Data were expressed as mean ± SD. Abbreviations: no sepsis – participants without sepsis development; early sepsis – participants who developed sepsis in first 72 h of life; late sepsis – participants who developed sepsis after 72 h of life; n.s. – non-significance.

In our further research, we tried to evaluate the concentrations of IFN-γ and IL-5 in cord blood and venous blood plasma. Taking into account that group 1 contains only three recruited participants, we focused to analyze mentioned cytokines in group 2 (32–36 weeks of GA) which enrolled 18 preterm infants. As shown in Figure 1, IFN-γ concentration in cord blood plasma did not significantly differ between sepsis groups (EOS and LOS) and control group (*p* > 0.05). Significantly higher levels (*p* < 0.001) of IFN-γ were observed only in control group between cord blood and venous plasma. Simultaneously, IFN-γ concentration in venous blood plasma significantly differs (*p* = 0.007) between EOS and LOS as well as between EOS and control groups (*p* < 0.001). Further, IFN-γ concentration in venous blood plasma showed significantly higher values in LOS (*p* < 0.001) compared to the control group of preterm infants (Figure 1).

**Figure 2:**
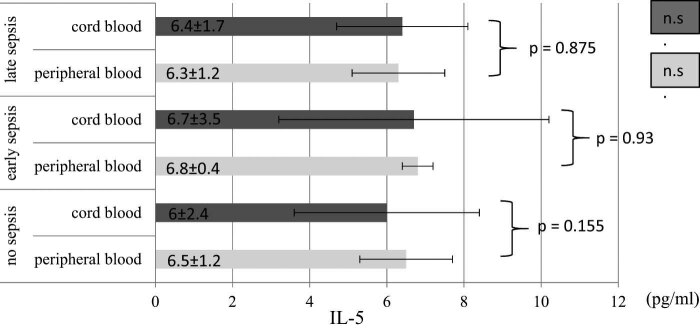
IL-5 (pg/mL) levels in the cord blood and peripheral blood among sepsis subgroups and control group. The plasma levels of IL-5 was evaluated as described in Material and methods section. Data were expressed as mean ± SD. Abbreviations: no sepsis – participants without sepsis development; early sepsis – participants who developed sepsis in first 72 h of life; late sepsis – participants who developed sepsis after 72 h of life; n.s. – non-significance.

On the other hand, concentration of IL-5 in cord blood and venous plasma did not significantly differ between sepsis groups (*P* > 0.05) as well as when sepsis and control groups of preterm infants are compared (Figure 2). Additionally, Figure 2 shows no statistical significant difference (*p* > 0.05) in IL-5 concentration (in cord blood and venous plasma) when compared to each group individually.

## 4 Discussion

Different human [9,10] and animal [11,12] studies have documented that sepsis provokes simultaneous release of numerous pro- and anti-inflammatory cytokines into the blood of premature infants. However, despite various studies, obtained results showed conflicting results about inflammatory mediators involved in sepsis prediction in premature neonates.

In our study, we tried to evaluate the role of representative cytokines of Th1 (IFN-γ) and Th2 (IL-5) immune response for sepsis prediction in premature neonates who are younger or older than 32 weeks of GA, mainly because named groups exhibit different levels of immune system maturity due to fetal development [2]. Taking into account that the difference in immune system maturity could be the origin of the heterogeneity of the immune response during sepsis [13], we also determined specific immune mediators (CRP and PCT) to have more elements for better prediction of sepsis development in preterm neonates [14]. Results obtained in our study demonstrated no statistical difference in sepsis (EOS and LOS) and control groups. Additionally, some clinical variables (birth weight, length, sex and Apgar score) showed no statistical significance when EOS and LOS were compared to the control group, indicating the equable manner of recruited participants and precisely inclusion of preterm neonates in our study. However, results from EOS group should be used with precaution, since this group includes seven participants, where only three of them developed sepsis. These results are in line with earlier reports [2,15], which enables more accurate evaluation of predictive factors for sepsis development in preterm neonates [16].

CRP is synthesized by the hepatocytes in a response to the infection, as a part of the innate immunity. Synthesis of this pentameric structure protein is stimulated by cytokines; and at the same time, it is the most commonly used test in the diagnosis of neonatal sepsis [17]. Our study results showed that CRP concentration in venous blood plasma did not significantly differ between two sepsis groups (EOS and LOS) and control group, indicating that this acute phase reactant protein may not be unreliable for early diagnosis of neonatal sepsis. The half-life of CRP is 24–48 h, and it needs around 12 h to reach increased level [18], which may in part clarify the results obtained in our study. Similar observations were reported previously, indicating that CRP evaluation 24–48 h after onset of symptoms has shown to increase its sensitivity for the diagnosis of neonatal sepsis [15]. Recent report confirmed the findings showing CRP tendency toward an increasing specificity after 24–48 h [19].

PCT is peptide prohormone of calcitonin and also an acute phase reactant protein. This protein is produced by hepatocytes and macrophages and has been shown to be associated with systemic inflammatory response syndrome (SIRS) [15]. Plasma levels of PCT in venous blood were markedly higher in sepsis groups (EOS and LOS) compared to the control group. The study also showed significantly elevated levels of PCT in EOS compared to the LOS group, suggesting that PCT plasma levels may represent potential useful marker for sepsis prediction. Similar results were obtained earlier, showing that elevated PCT levels can be appropriate for systemic fetal inflammatory response [20,21]. Elevated PCT levels occur within 2–4 h following the bacterial endotoxin exposure and remain increased for the next 24 h [22]. Even the half-life of PCT is 24–30 h, the rapid increase in PCT with the onset of bacterial infection makes it a proper marker for early diagnosis of neonatal sepsis [15], which corresponds to our study results. Furthermore, the observations were confirmed in previously reported meta-analysis which showed PCT as a helpful biomarker for early diagnosis of sepsis [23].

Cell-mediated immunity involves two main types of T cells, including cytotoxic T lymphocytes (CD8+) and T-helper lymphocytes (CD4+). CD8+ cells are involved in the eradication of intracellular pathogens while CD4+ cells are further divided into Th1 and Th2 CD4+ cells mainly defined by their cytokine profile. Th1 cells produce inflammatory cytokines (IFN-γ, IL-2 and TNFα), while anti-inflammatory cytokines (Il-4, IL-5, IL-13 and IL-10) are mainly produced by Th2 cells [24]. IFN-γ is a soluble cytokine secreted by Th1 cells, macrophages, NK cells and mucosal epithelial cells [25]. In our study, we found that IFN-γ plasma levels were notably elevated in EOS and LOS groups compared to the control group of preterm infants as well as in EOS group when compared with LOS group. On the other hand, IFN-γ concentrations in cord blood plasma did not significantly differ among the evaluated groups of preterm neonates. These findings may indicate that the levels of plasma IFN-γ concentrations, rather than levels of IFN-γ in cord blood, could be better predictive factor of sepsis development in preterm neonates. In line with our results are earlier report confirming that IFN-γ induces upregulation of Toll-like receptors and stimulates phagocytosis [26]. Similar results were confirmed in earlier studies [2,16,27] while some reports documented decreased IFN-γ levels in preterm neonates [28,29]. The reason for these conflict results are still unclear. Some factors have the ability to affect cytokine concentrations, including GA, sepsis definition (clinical sepsis or proven culture) or including various microorganisms (which may elicit different cytokine response) [16]. Increased IFN-γ levels in cord blood plasma, compared to venous blood, were only observed in preterm neonates without sepsis development. Observed alterations of cytokine levels in intrauterine growth has been shown in different reports, but functional significance of these findings needs to be clarified [30]. Our study results showed that IL-5 levels did not significantly differ between evaluated groups, as well as we compared IL-5 levels in cord and venous blood, suggesting that IL-5 may not be credible predictor for sepsis development in preterm neonates. IL-5 represents cytokine produced by Th2 and mast cells. This cytokine stimulates B cell growth, increases immunoglobulin secretion and stimulates eosinophil activation [31]. Preterm neonates have deficient T cell function due to greater proportion of naïve T cell. Also, in preterm neonates class-switch to express another antibody is reduced, as well as production of total amount of antibodies [24]. Furthermore, during fetal life, cytokine responses are mainly driven towards a Th2 phenotype. Due to bias to Th2 phenotype, preterm neonates are vulnerable to infection. It is believed that rapid switch towards Th1 phenotype and to production of Th1 cytokines may be one of the defensive mechanisms of preterm neonates [24,32], which may explain elevated IFN-γ levels and unchanged IL-5 levels observed in our study. Concentrations of IFN-γ varies depending on the time of sepsis and GA of preterm neonates [2] and this, also, should be considered when this cytokine is used for sepsis prediction.

In summary, we have shown that evaluation of IFN-γ levels, rather from venous blood than cord blood, have more predictive potential for sepsis development in preterm neonates. On the other hand, IL-5 level and CRP values did not show a predictive value for early detection of sepsis development in preterm neonates. Evaluation of PCT levels in venous blood may represent as a helpful factor, together with IFN-γ levels, for sepsis prediction in preterm neonates. However, further studies, including larger number of cohorts and preterm neonates matched for GA are needed to get more insight in prediction of sepsis development among preterm neonates.
